# Beneficial Effects of Dinitrosyl Iron Complexes on Wound Healing Compared to Commercial Nitric Oxide Plasma Generator

**DOI:** 10.3390/ijms24054439

**Published:** 2023-02-23

**Authors:** Alexandra Igrunkova, Alexey Fayzullin, Natalia Serejnikova, Tatiana Lipina, Alexandr Pekshev, Anatoly Vanin, Victoria Zaborova, Elena Budanova, Dmitry Shestakov, Igor Kastyro, Anatoly Shekhter

**Affiliations:** 1 Department of Human Anatomy and Histology, N.V. Sklifosovsky Institute of Clinical Medicine, Sechenov First Moscow State Medical University (Sechenov University), Moscow 119991, Russia; 2 Department of Experimental Morphology and Biobanking, Institute for Regenerative Medicine, Sechenov First Moscow State Medical University (Sechenov University), Moscow 119991, Russia; 3 World-Class Research Center “Digital Biodesign and Personalized Healthcare”, Sechenov First Moscow State Medical University (Sechenov University), Moscow 119991, Russia; 4 Department of Cell Biology and Histology, Biological Faculty, Moscow Lomonosov State University, Moscow 119234, Russia; 5 Research Institute of Power Engineering, Bauman Moscow State Technical University, Moscow 105005, Russia; 6 Semenov Federal Research Center of Chemical Physics, Russian Academy of Sciences, Chernogolovka 142432, Russia; 7 Department of Sports Medicine and Medical Rehabilitation, N.V. Sklifosovsky Institute of Clinical Medicine, Sechenov First Moscow State Medical University (Sechenov University), Moscow 119991, Russia; 8 Laboratory of Sports Adaptology, Moscow Institute of Physics and Technology, National Research University, Dolgoprudny 141700, Russia; 9 Department of Microbiology, Virology and Immunology Named after A.A. Vorobiev, F. Erismann Institute of Public Health, Sechenov First Moscow State Medical University (Sechenov University), Moscow 119991, Russia; 10 Moscow Clinical Scientific Center Named after A. S. Loginov, Moscow 111123, Russia; 11 Medical Institute, People’s Friendship University of Russia (RUDN University), Moscow 117198, Russia

**Keywords:** wound healing, nitric oxide, nitric oxide donors, dinitrosyl iron complexes (DNIC), NO-containing gas flow, Plason device, NO-therapy

## Abstract

Nitric oxide (NO) is a gaseous molecule which plays a key role in wound healing. Previously, we identified the optimal conditions for wound healing strategies using NO donors and an air plasma generator. The aim of this study was to compare the wound healing effects of binuclear dinitrosyl iron complexes with glutathione (B-DNIC-GSH) and NO-containing gas flow (NO-CGF) at their optimal NO doses (0.04 mmol for B-DNIC-GSH and 1.0 mmol for NO-CGF per 1 cm^2^) in a rat full-thickness wound model over a 3-week period. Excised wound tissues were studied by light and transmission electron microscopy and immunohistochemical, morphometrical and statistical methods. Both treatments had an identical stimulating impact on wound healing, which indicated a higher dosage effectiveness of B-DNIC-GSH compared to the NO-CGF. B-DNIC-GSH spray application reduced inflammation and promoted fibroblast proliferation, angiogenesis and the growth of granulation tissue during the first 4 days after injury. However, prolonged NO spray effects were mild compared to NO-CGF. Future studies should determine the optimal B-DNIC-GSH solution course for a more effective wound healing stimulation.

## 1. Introduction

Nitric oxide (NO) is an endogenous gaseous signaling molecule which regulates multiple biological functions, including vasospasm, transmission of nerve impulses and inflammation. It has been thoroughly demonstrated that NO plays a key role in wound healing [1,2,3]. The application of exogenous NO became a popular trend in wound therapy based on the fact that insufficient NO tissue concentration disrupts regeneration [4]. NO application was shown to be beneficial in a range of medical conditions varying from traumatic and diabetic wounds to acute respiratory distress syndrome [1,5,6,7].

There are two main NO-therapy directions used for wound healing stimulation: NO containing plasma and pharmaceutical NO donors or NO-synthase inductors [4,8,9,10]. The variety of NO donors and their ability to be locally delivered as components of implantable biomaterials and tissue-engineered constructs make them the most promising candidates for NO-therapy [4,11].

Dinitrosyl iron complex (DNIC) is the primary storage form of NO in organisms. Its mononuclear form (M-DNIC) ((RS-)2Fe^2+^(NO)(NO^+^)) consists of an iron atom, two thiol residues and two nitrosyl ligands, namely, the neutral NO molecule and the nitrosonium cation (NO^+^) [10,11]. DNIC has been broadly studied in cardiology, oncology and pulmonology. The clinical trials of Oxacom (DNIC with a glutathione ligand) demonstrated its safety and pronounced hypotensive effect when administered intravenously at a dose of 1 mg/kg/min [12,13]. DNIC induced tumor cell apoptosis in experiments on prostate cancer cell lines (PC-3), breast cancer (SKBR-3) and non-small cell lung cancer (CRL5866) [14]. In vivo studies have also confirmed the efficacy of DNIC on microvasculature growth control in oncology [15]. Burgova et al. demonstrated that intraperitoneal injections of DNIC with thiol ligands at a dose of 20 µM/kg for 10–12 days completely suppressed the growth of endometriosis tumor nodes [16].

Several research groups have reported that DNIC administration, primarily in the form of wound bottom injections, can accelerate wound healing [11,17,18,19,20]. However, these injections are associated with local traumatization and systemic complications. To solve this problem, we developed a new delivery form of DNIC (spray) and determined the most effective dose for facilitation of wound healing. We identified the most effective dosage for spray with binuclear dinitrosyl iron complexes with glutathione (B-DNIC-GSH, formula ((GS^−^)_2_Fe^2+^_2_(NO)_2_(NO^+^)_2_)), which was 16.6 mg/cm^2^ (0.02 mmol B-DNIC-GSH per 1 cm^2^ calculated in relation to one atom of iron in this nuclear complex) [19]. Since each iron atom in the compound is bound to two nitrosyl ligands, a maximum of 0.04 mmol NO could be released in gaseous form from 0.02 mmol B-DNIC-GSH. This dosage of B-DNIC-GSH inhibited inflammation and promoted the growth and maturation of granulation tissue by day 4 of wound healing.

Plasma medicine is a rapidly developing field utilizing plasma which contains a varying range of active components with pro-regenerative effects, including inert gas molecules, ions and oxides. The dermatology and cosmetology markets have created demand for medical plasma devices: kINPen MED (INP Greifswald/neoplas tools GmbH, Greifswald, Germany), PlasmaDerm VU-2010 (CINOGY Technologies GmbH, Duderstadt, Germany), PlasmaCare (Terraplasma Medical, Garching, Germany), SteriPlas (Adtec Ltd., London, UK) and PlasmaJet (Plasma Surgical Ltd., Atlanta, GA, USA) [9,21,22]. Plason generates high concentrations (up to ~5000 ppm) of nitric oxide and small amounts of by-products, including Nitrogen Dioxide (NO_2_), OH and H_2_O_2_, in comparison to the other devices [22]. This unique feature of the Plason defines the treatment effects related to NO mechanisms of action.

It has been used in clinical practice since 2000 to treat wounds, trophic and diabetic ulcers, burns, cornea injuries, sports injuries, arthritis and other pathologies [1,23]. The bulk of clinical evidence allows us to use it as a commercial control in our study.

We demonstrated in our previous studies that effective treatment of skin wounds with NO-CGF generated by the Plason device was achieved by applying 30 mg (1 mmol) of NO per 1 cm^2^ of wound surface [8]. This value, in a millimolar ratio, was 25 times higher than the above dose of NO released from B-DNIC-GSH for the optimal healing effect. This fact may indicate the high efficiency of these complexes as compounds that accelerate the healing of skin wounds. However, before making such a conclusion, it was necessary to investigate whether the wound healing effects of these agents would be the same in all morphological, biochemical and physiological parameters at the specified optimal doses. This hypothesis was tested in the present work.

## 2. Results

### 2.1. Gross Examination

**Figure 1:**
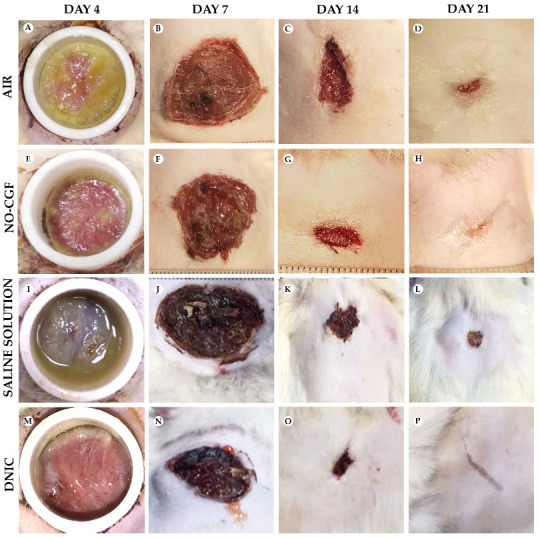
Gross examination of wounds in animals on post-operative days 4 (**A**,**E**,**I**,**M**), 7 (**B**,**F**,**J**,**N**), 14 (**C**,**G**,**K**,**O**) and 21 (**D**,**H**,**L**,**P**).

On Day 4, the wound surfaces in the control groups (air, saline) were covered with a relatively thick layer of loose fibrin and exudate (Figure 1A,I). Fibrin accumulation was observed along the wound edges in three animals treated with NO-CGF (Figure 1E). In the DNIC group, a thin layer of exudate covered the wound bottoms, and a loose whitish film of fibrin was observed along the wound edges in two rats (Figure 1M). Moderate exudation and fibrin production indicated development of the inflammatory phase of wound healing. The other animals of the experimental groups had smooth and shiny wound surfaces.

**Figure 2:**
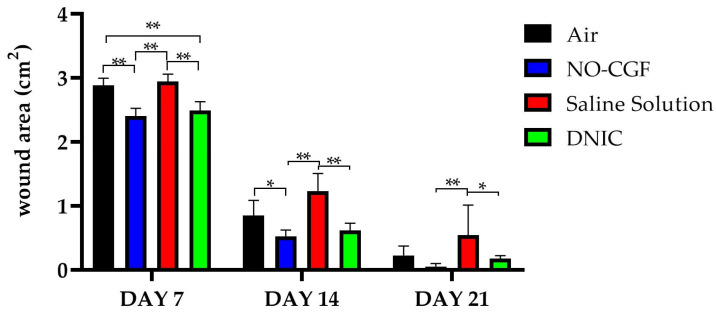
Wound area dynamics, two-way ANOVA, mean values ± SD. *—*p* < 0.05, **—*p* < 0.01.

On Day 7, all the wounds were covered with dense fibrin clots with a rough surface; they were tightly bound to the underlying tissues (Figure 1B,F,J,N). The animals in the experimental groups had the smallest wound areas (2.4 ± 0.12 cm^2^ for NO-CGF and 2.5 ± 0.14 cm^2^ for DNIC). They were significantly smaller than in the saline control group (2.94 ± 0.12 cm^2^) (*p* < 0.01) or air control group (2.9 ± 0.11 cm^2^) (*p* < 0.01) (Figure 2).

On Day 14, we revealed a pronounced marginal epithelialization and wound area reduction in the experimental groups. The wound bottoms of the animals of the control groups were clean, except for one case of purulent inflammation in the saline control group (Figure 1C,K). The areas of the NO treated wounds were significantly smaller than in the saline control group (0.52 ± 0.10 cm^2^ for NO-CGF and 0.61 ± 0.12 cm^2^ for DNIC against 1.23 ± 0.27 cm^2^ for saline) (*p* < 0.01) (Figure 1G,O and Figure 2).

By Day 21, the wounds were completely epithelialized in five out of six animals of the NO-CGF group and four out of six rats in the DNIC group (Figure 1H,P). The wound areas in these groups, especially where NO-CGF was applied, were statistically smaller compared to the saline control group (*p* < 0.05) (Figure 1H,L and Figure 2). In the air control group, complete epithelialization was observed in three animals (Figure 1D).

### 2.2. Histological Study

**Figure 3:**
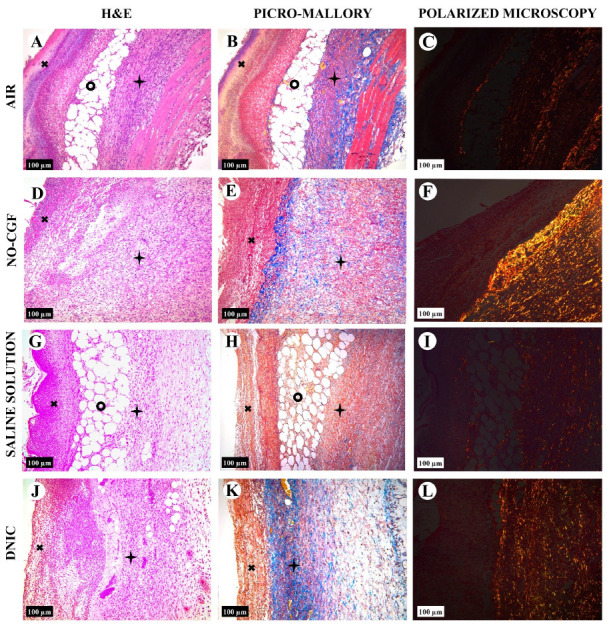
Histology of the wounds at post-operative day 4, hematoxylin and eosin staining (**A**,**D**,**G**,**J**), picro-Mallory staining (**B**,**E**,**H**,**K**) staining and polarized light microscopy; picrosirius red staining (**C**,**F**,**I**,**L**), scale bar—100 µm. 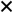
—fibrin clot, 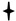
—granulation tissue, 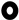
—adipose tissue.

**Figure 4:**
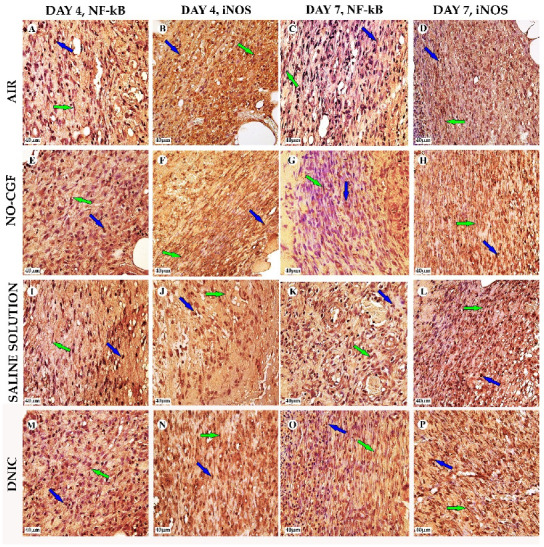
NF-κß (**A**,**B**,**E**,**F**,**I**,**J**,**M**,**N**) and iNOS (**C**,**D**,**G**,**H**,**K**,**L**,**O**,**P**) expression in the study groups on days 4 and 7, magnification ×400. Green arrow—fibroblast, blue—macrophage. (**A**) NF-κß cytoplasmic expression in macrophages, (**B**) iNOS expression in fibroblasts of immature granulation tissue, (**C**) weak cytoplasmic expression of NF-κβ in fibroblasts, (**D**) iNOS-positive cells in the superficial part of wound, (**E**) intensive nuclear NF-κß expression in macrophages, (**F**) iNOS-positive fibroblasts in the deep layers of wounds, (**G**) NF-κβ expression in fibroblasts, (**H**) parallel orientated iNOS-positive fibroblasts, (**I**) weak NF-κß-positive cytoplasmic expression, (**J**) iNOS expression in macrophages, (**K**) NF-κß nuclear expression in chaotically oriented cells, (**L**) intensive iNOS expression, (**M**) prevalence of cells with nuclear expression, (**N**) intensive diffuse iNOS expression, (**O**) intensive NF-κβ cytoplasmic expression in fibroblasts and (**P**) numerous iNOS-positive macrophages.

**Figure 5:**
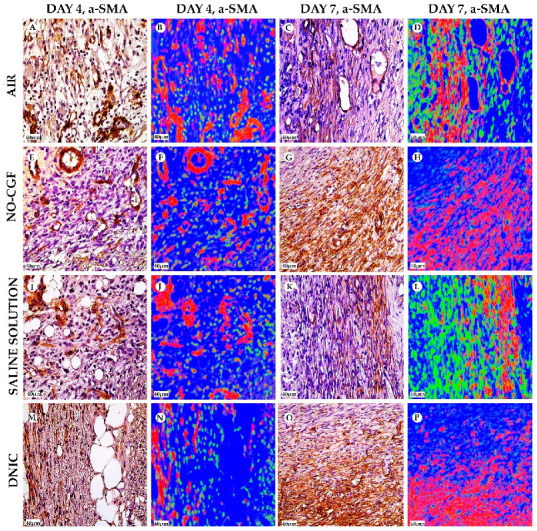
α-SMA expression and α-SMA-positive cells’ distribution in the study groups on days 4 (**A**,**B**,**E**,**F**,**I**,**J**,**M**,**N**) and 7 (**C**,**D**,**G**,**H**,**K**,**L**,**O**,**P**), magnification ×400. Red—vimentin-positive cells, green—vimentin-negative cells, blue—background. (**A**) α-SMA expression in endothelial cells, (**B**) well-detected contours of newly formed capillaries, (**C**) cytoplasmic α-SMA expression in the superficial part of the granulation tissue, (**D**) thin layer of red-stained cells, (**E**) singular spindle-shaped α-SMA-positive cells, (**F**) predominance of green-stained cells, (**G**) diffuse α-SMA expression, (**H**) numerous parallel orientated red cells, (**I**) immature granulation tissue with α-SMA expression in endothelium, (**J**) chaotically arranged blood vessels with red-stained walls, (**K**) α-SMA-positive cells in deep part of wound, (**L**) thin layer of positive-stained cells, (**M**) α-SMA-positive cells along the fibrin fibers and (**N**) red-stained cells in superficial part of wound, (**O**) diffuse α-SMA staining in the deep part of wound, (**P**) a thick layer of α-SMA-positive cells.

**Figure 6:**
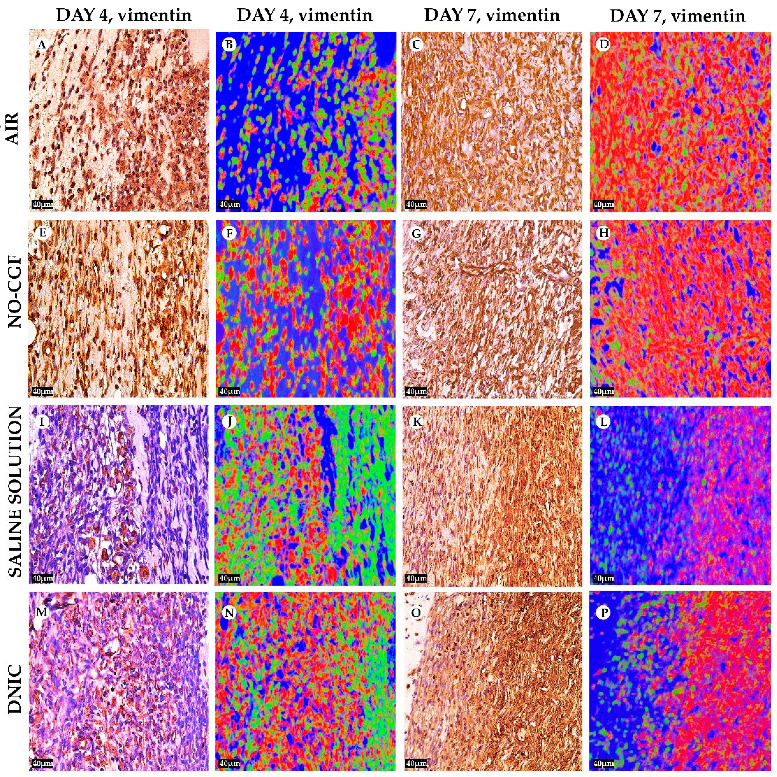
Vimentin expression and vimentin-positive cells distribution in the study groups on days 4 (**A**,**B**,**E**,**F**,**I**,**J**,**M**,**N**) and 7 (**C**,**D**,**G**,**H**,**K**,**L**,**O**,**P**), magnification ×400. Red—vimentin-positive cells, green—vimentin-negative cells, blue—background. (**A**) Cells intensively express vimentin, (**B**) positive cells occupy a small area, (**C**) numerous vimentin-positive spindle-shaped cells, (**D**) diffusely arranged red-stained cells, (**E**) vimentin-positive cells orientate parallel to wound surface, (**F**) prevalence of positive spindle cells, (**G**) prevalence of vimentin-positive cells, (**H**) a layer of red-stained cells, (**I**) predominant vimentin expression in pericytes, (**J**) singular positive cells in granulation tissue, (**K**) vimentin-positive cells in the deep part of wound, (**L**) a layer of red-stained cells in the deep part of wound, (**M**) vimentin expression in chaotically arranged fibroblasts, (**N**) high content of vimentin-positive (red-stained) cells, (**O**) intensive diffuse vimentin expression and (**P**) a layer of vimentin expressing cells.

**Figure A1:**
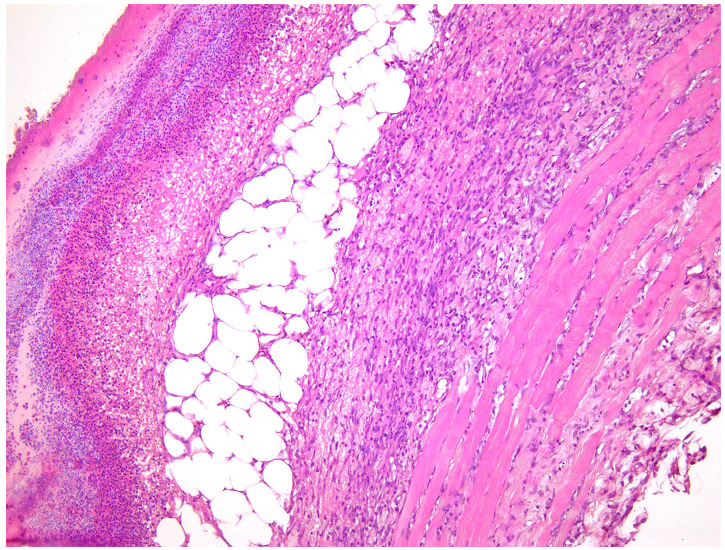
Histology of the wound treated with air at post-operative day 4, hematoxylin and eosin staining, magnification—10×.

On Day 4, thick fibrin clots covered the wound bottoms in the air control group (Figure 3A and Figure A1 Appendix A). Microbial colonies were observed in fibrin clots in two out of six animals. Under them, islets of immature granulation tissue consisted of chaotically orientated vimentin and alpha smooth muscle actin (α-SMA) positive cells, inducible Nitric Oxide synthase (iNOS) positive fibroblasts, singular capillaries, diffuse immune cell infiltration with neutrophils and Nuclear factor kappa B (NF-κß) positive macrophages (Figure 3B, Figure 4A,B, Figure 5A,B and Figure 6A,B). Collagen fibers had intensive anisotropy in the deep wound area (Figure 3C).

**Figure 7:**
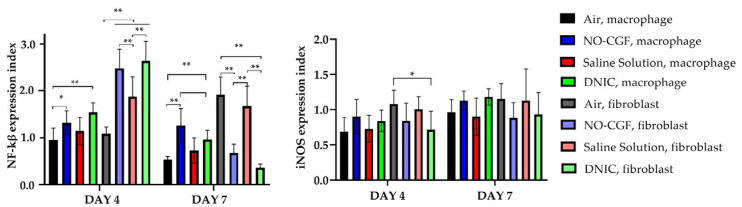
Activity index of NF-κß and iNOS. Two-way ANOVA, median ± 95% CI. *—*p* < 0.05, **—*p* < 0.01.

**Figure A2:**
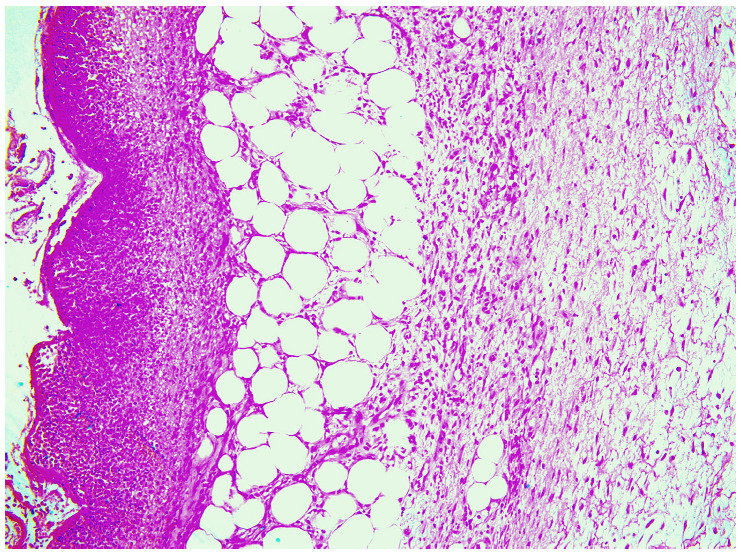
Histology of the wound treated with NO-CGF at post-operative day 4, hematoxylin and eosin staining, magnification—10×.

In the NO-CGF group, the signs of inflammation were moderate and microbial contamination was absent (Figure 3D and Figure A2). NF-κß and iNOS expression in macrophages and fibroblasts was more intensive compared with the air control group (*p* < 0.05) (Figure 4E,F and Figure 7). The collagen fibers were orientated along the wound bottom. The tissue was rich with proliferating vimentin and α-SMA-expressing cells, and the blood vessels were stretched between the wound surface and deeper layers of soft tissues (Figure 3E, Figure 5E,F and Figure 6E,F). Anisotropy of granulation tissue fibers was low (Figure 3F).

In the saline control group, the fibrin clots were thick, and they contained bacterial colonies. The granulation tissue was characterized by a low content of collagen fibers, α-SMA-positive cells, vasculitis and diffuse lymphocyte and iNOS-positive macrophage infiltration (Figure 3G,H, Figure 4J and Figure 5I,J). Fibroblasts were vimentin positive and expressed NF-κß-positive in cytoplasm (Figure 4I and Figure 6I,J). Polarized light microscopy revealed thin collagen fibers with green anisotropy (Figure 3I).

**Figure 8:**
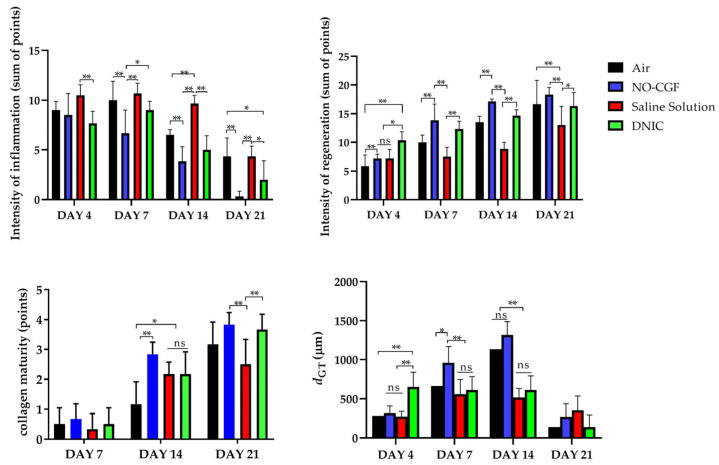
Morphometry of the wound tissues, two-way ANOVA tests, median and 95% CI. dGT—granulation tissue thickness, ns—non-significant, *—*p* < 0.05; **—*p* < 0.01.

**Figure 9:**
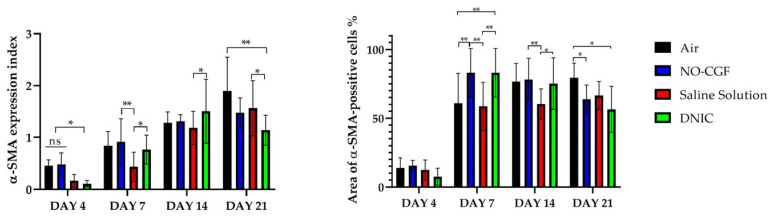
α-SMA expression index and area of α-SMA-positive cells, two-way ANOVA, mean values ± SD; ns—no differences, *—*p* < 0.05, **—*p* < 0.01.

In the DNIC group, the fibrin clots were thin and contained singular bacterial colonies. Immune cell infiltration with iNOS-positive macrophages and lymphocytes and microcirculatory disorders were less pronounced (Figure 3J and Figure 4N). The inflammatory index showed a significantly lower intensity than in the previous group (*p* < 0.01) (Figure 6). In this group, the NF-κß activity index was 42% higher than in the air control group (*p* < 0.01) and 27% higher than in the saline control group (*p* < 0.01) (Figure 4M and Figure 7). The granulation tissue layer was 57% thicker than in the air control group and 59% thicker than in the saline control group (Figure 8). It consisted of vertically oriented capillaries and densely packed vimentin-positive fibroblasts (Figure 3J and Figure 6M,N). Multiple collagen fibers were stained blue by picro-Mallory (Figure 3K). The α-SMA expression index was lower than in the groups where the wounds were blown with NO-CGF or air (*p* < 0.05) (Figure 5M,N and Figure 9). Polarized light microscopy revealed the predominance of yellow glowing of collagen fibers in the deep wound areas (Figure 3L). These signs suggest the maturation of the granulation tissue.

**Figure 10:**
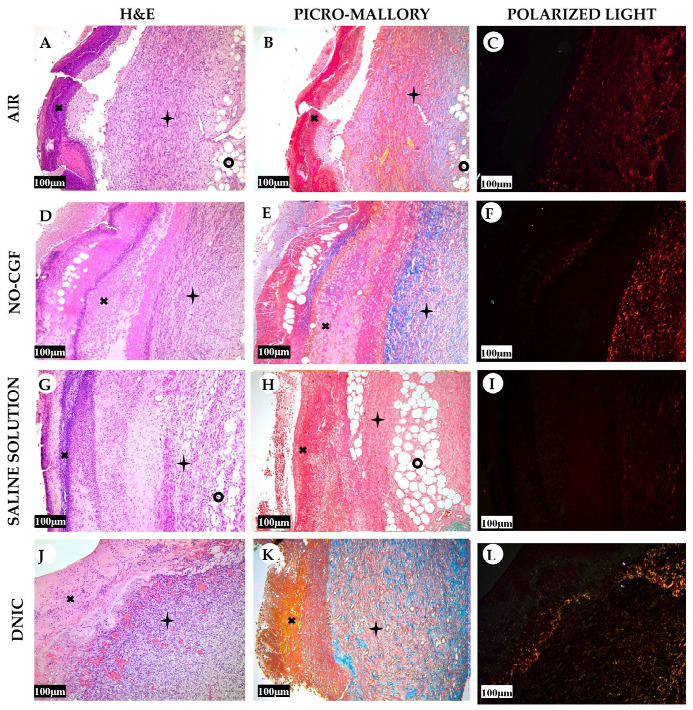
Histology of the wounds at post-operative day 7, hematoxylin and eosin staining (**A**,**D**,**G**,**J**), picro-Mallory staining (**B**,**E**,**H**,**K**) staining and polarized light microscopy (**C**,**F**,**I**,**L**); scale bar—100 μm. 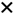
—fibrin clot, 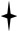
—granulation tissue, 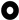
—adipose tissue.

**Figure 11:**
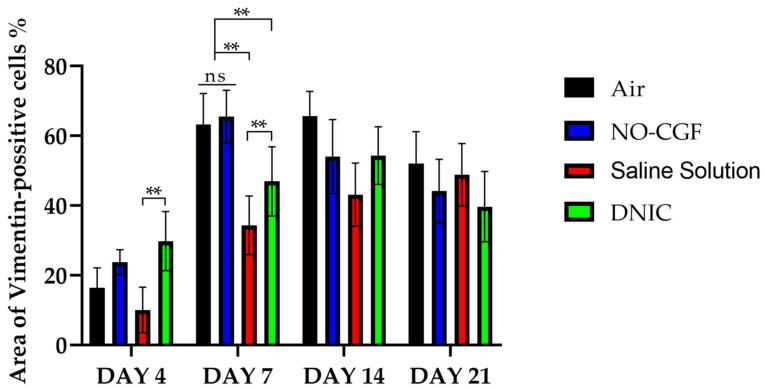
Area of vimentin-positive cells, two-way ANOVA, mean values ± SD; ns—non-significant, **—*p* < 0.01.

On Day 7, we observed marginal epithelization of wounds in all study groups. Central parts of the wounds were covered with fibrin clots. The thickest fibrin clot was in the air control group. The granulation tissue comprised fibroblasts with NF-κβ cytoplasmic expression, mild diffuse lymphocyte and iNOS-positive macrophage infiltration, and many congested capillaries, with productive endo- and panvasculitis (Figure 4C,D and Figure 10A). The area of cells with cytoplasmic α-SMA expression was significantly lower compared to the groups in which wounds were treated with NO (*p* < 0.01) (Figure 5C,D and Figure 9). Collagen fibers were found only in deep wound areas (Figure 10B). They had low anisotropy and maturity (Figure 10C). In this group, the mean area of vimentin-positive cells was 63.25% (±8.75), which was almost 1.5-fold higher than in the DNIC group (*p* < 0.01) and about 2-fold higher than in the saline control group (*p* < 0.01) (Figure 6C,D and Figure 11).

We found bacterial contamination in only one animal of the group treated with NO-CGF. The fibrin clot thickness was thinner than in the previous group. Half of the animals had immature granulation tissue, while it was mature in the rest (Figure 10D). Spindle-shaped fibroblasts intensively expressed iNOS (Figure 4H). Vimentin-positive cells (fibroblasts) occupied the largest area relative to the other groups (65.5% (±7.55)), and the area of α-SMA-positive cells was 53% larger than in the saline control group (Figure 5G,H, Figure 6G,H, Figure 9 and Figure 11). Collagen fibers were arranged in parallel, and capillaries were oriented vertically toward the wound surface. The NF-κß activity index in fibroblasts was significantly higher than in the control groups (air, saline) (*p* < 0,01) (Figure 4G and Figure 7). Picro-Mallory staining showed the prevalence of collagen fibers in this group compared to the air control group (Figure 10E). Polarized light microscopy revealed the red color of collagen fibers, mainly in the deep layers of the wound (Figure 10F).

**Figure A3:**
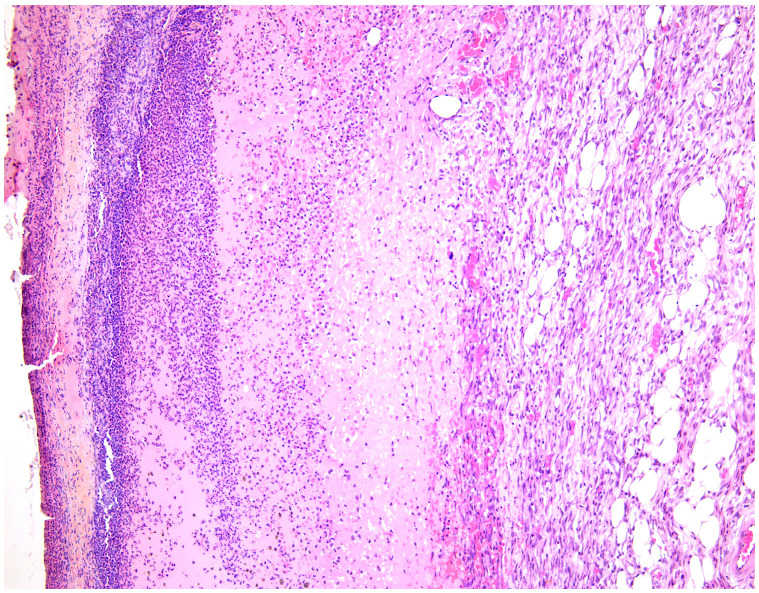
Histology of the wound treated with saline solution at post-operative day 7, hematoxylin and eosin staining, magnification—10×.

In the saline control group, the granulation tissue was very immature in four out of six cases. It was characterized by the moderate proliferation of iNOS and NF- κß-positive fibroblasts, poor angiogenesis, lymphostasis and diffuse infiltration with immune cells (Figure 4K,L, Figure 10G and Figure A3). The α-SMA expression index and the area of vimentin-positive cells was minimum (mean 0.43 ± 2.8) (Figure 5K,L, Figure 6K,L, Figure 9 and Figure 11). Picro-Mallory staining revealed numerous collagen fibers of dark blue color, which had very weak birefringence in polarized light microscopy (Figure 10H,I).

**Figure A4:**
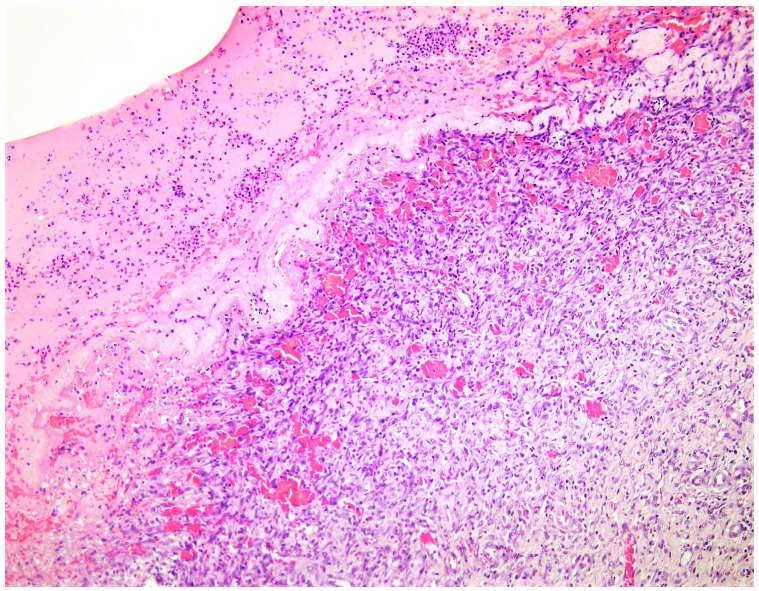
Histology of the wound treated with DNIC at post-operative day 7, hematoxylin and eosin staining, magnification—10×.

The DNIC spray application reduced the severity of exudation, inflammatory infiltration and microcirculatory disorders more effectively related to the other groups. The number of macrophages decreased, but they expressed iNOS 30% more actively than in the saline control group (*p* < 0.05) (Figure 4P and Figure 7). The granulation tissue’s maturity was higher compared to Day 4, but the volume did not change significantly (Figure 8, Figure 10J and Figure A4). The area of α-SMA-positive cells was higher by 43% than in the saline control group (*p* < 0.05) (Figure 5O,P and Figure 9). Vimentin-positive cells (fibroblasts) with intensive NF-κβ cytoplasmic expression were orientated in parallel to the wound surface and actively produced collagen fibers, which was detected by picro-Mallory staining (Figure 4O, Figure 6O,P and Figure 10K). Under polarized light microscopy, thin, newly-formed collagen appeared green and yellow in color (Figure 10L).

**Figure 12:**
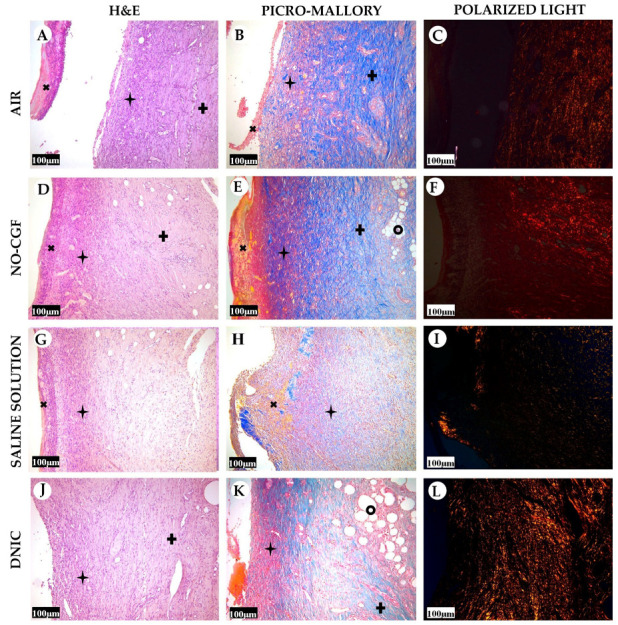
Histology of the wounds at post-operative day 14, hematoxylin and eosin staining (**A**,**D**,**G**,**J**), picro-Mallory staining (**B**,**E**,**H**,**K**) staining and polarized light microscopy (**C**,**F**,**I**,**L**); scale bar—100 μm. 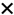
—fibrin clot, 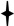
—granulation tissue, 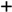
—fibrotic tissue, 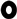
—adipose tissue.

**Figure 13:**
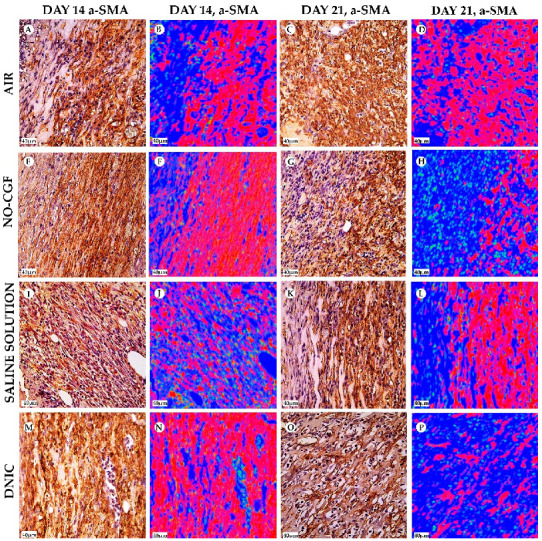
α-SMA expression and α-SMA-positive cells distribution in the study groups on days 14 (**A**,**B**,**E**,**F**,**I**,**J**,**M**,**N**) and 21 (**C**,**D**,**G**,**H**,**K**,**L**,**O**,**P**), magnification ×400. Red—α-SMA-positive cells, green—α-SMA-negative cells, blue—background. (**A**) α-SMA expression in deep part of wound, (**B**) thick layer of red-stained cells, (**C**) diffuse α-SMA expression, (**D**) monolayer of red-stained cells, (**E**) α-SMA-positive spindle-shaped cells, (**F**) dense layer of red-stained cells, (**G**) SMA-positive cells in deep part of wound, (**H**) low content of red-stained cells, (**I**) α-SMA-positive cells in granulation tissue, (**J**) parallel orientated red-stained cells, (**K**) intensive α-SMA expression, (**L**) a layer of red-stained cells in deep part of wound, (**M**) numerous α-SMA-positive cells along collagen fibers, (**N**) dense red-stained layer, (**O**) α-SMA expression in endothelial cells and (**P**) singular red-stained cells.

**Figure 14:**
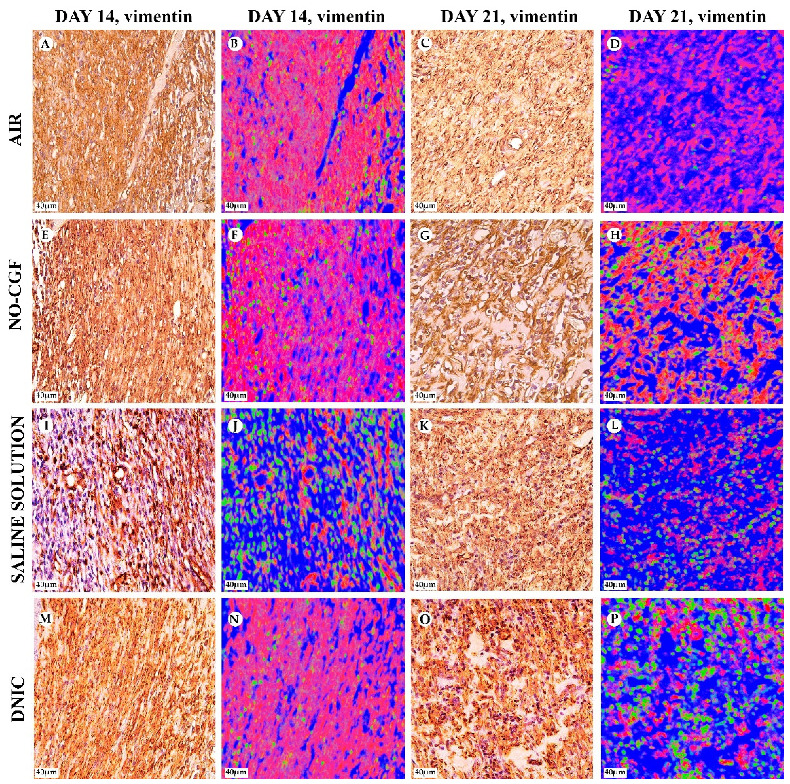
Vimentin expression and vimentin-positive cells’ distribution in the study groups on days 14 (**A**,**B**,**E**,**F**,**I**,**J**,**M**,**N**) and 21 (**C**,**D**,**G**,**H**,**K**,**L**,**O**,**P**), magnification ×400. Red—vimentin-positive cells, green—vimentin-negative cells, blue—background. (**A**) Numerous vimentin-positive cells, (**B**) monolayer of red-stained cells, (**C**) weak vimentin expression, (**D**) red-stained areas in mature granulation tissue, (**E**) intensive vimentin expression, (**F**) high content of vimentin-positive cells in mature granulation tissue, (**G**) vimentin-positive spindle-shaped cells along collagen fibers, (**H**) prevalence of red-stained cells, (**I**) vimentin expression in the deep part of wound, (**J**) singular positive cells in granulation tissue, (**K**) low intensity of vimentin expression, (**L**) diffusely arranged red-stained cells, (**M**) prevalence of positive spindle-shaped cells, (**N**) high content of red-stained cells, (**O**) vimentin expression in pericytes and fibroblasts and (**P**) singular red-stained areas.

**Figure A5:**
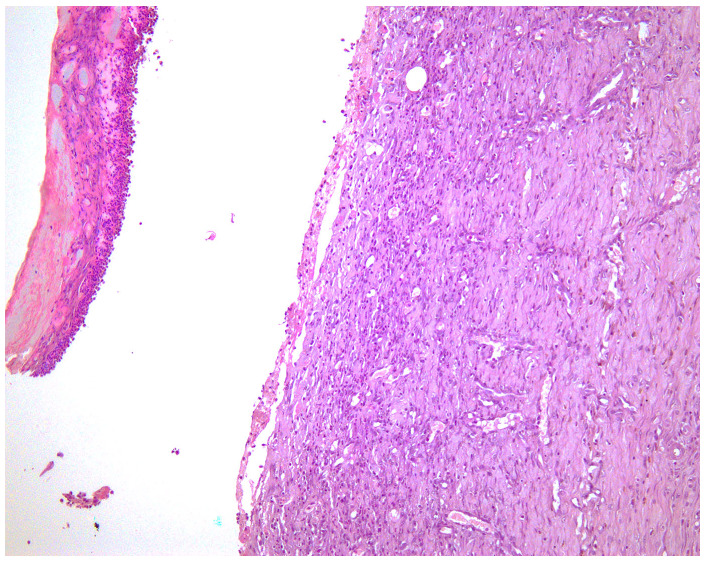
Histology of the wound treated with air at post-operative day 14, hematoxylin and eosin staining, magnification—10×.

On Day 14, in the air control group, the fibrin clot covered the wound’s center, while epithelialization was detected only along the edges. Mature granulation tissue comprised thick collagen fibers (dark blue by picro-Mallory), numerous α-SMA-positive cells (myofibroblasts), singular blood vessels and spindle-shaped fibroblasts in the deep layers of the wound (Figure 12A,B, Figure 13A,B and Figure A5). In this group, vimentin-positive cells occupied the maximum area (Figure 11 and Figure 14A,B). The cells were located in parallel and close to each other. The collagen had low maturity and anisotropy in polarized light microscopy (Figure 12C).

**Figure A6:**
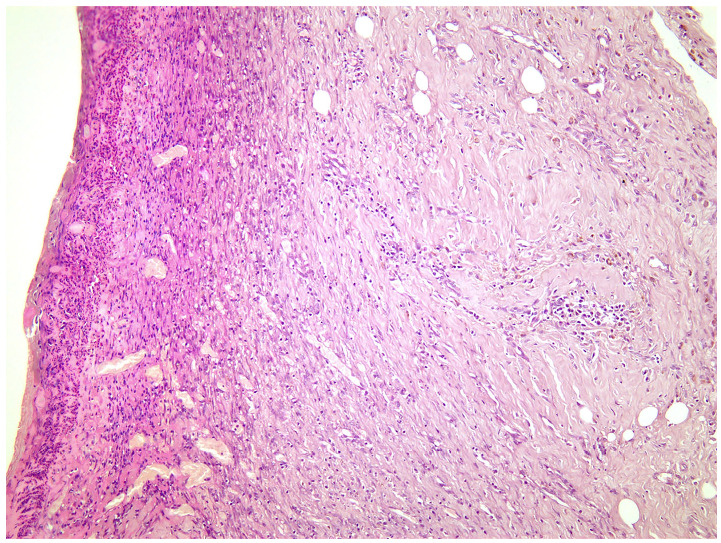
Histology of the wound treated with NO-CGF at post-operative day 14, hematoxylin and eosin staining, magnification—10×.

A highly differentiated epithelium completely covered the wound’s surface in three out of six animals from the NO-CGF group. Scar tissue was observed along the wound’s periphery (Figure 12D and Figure A6). The area of α-SMA-positive cells in wound centers was evidently larger than in the saline control group (*p* < 0.01) (Figure 9 and Figure 13E,F). We revealed a high content of collagen fibers; they were stained dark blue by picro-Mallory and had a red color in polarized light microscopy (Figure 12E,F). The number of fibroblasts decreased in the granulation tissue as it matured. Thus, the cells encompassed a smaller area than on Day 7, despite intensive vimentin expression (Figure 6 and Figure 14E,F).

In the saline control group, the wound centers were covered with a dense fibrin clot, and immune cell infiltration and microcirculatory disorders were observed in two out of six animals. The granulation tissue volume and maturity were minimal: the wound contained few α-SMA and vimentin-positive spindle-shaped cells (Figure 12G, Figure 13I,J and Figure 14I,J). Picro-Mallory staining demonstrated a relatively low content of collagen fibers (Figure 12H). They were birefringent and characterized by pale green and yellow color (Figure 12I).

The wound surfaces were completely epithelized in two out of six animals from the DNIC group. The granulation tissue was less mature than in the NO-CGF group (Figure 12J). It comprised vimentin-positive fibroblasts synthesizing collagen fibers parallel to the wound and capillaries with perpendicular orientation to the wound surface (Figure 12K and Figure 14M,N). The α-SMA expression index was higher than in the saline control group (*p* < 0.05) (Figure 9 and Figure 13M,N). Polarized light microscopy revealed red-stained fibers along the periphery along with yellow and green fibers in the wound center, which indicated collagen maturation (Figure 12L).

**Figure 15:**
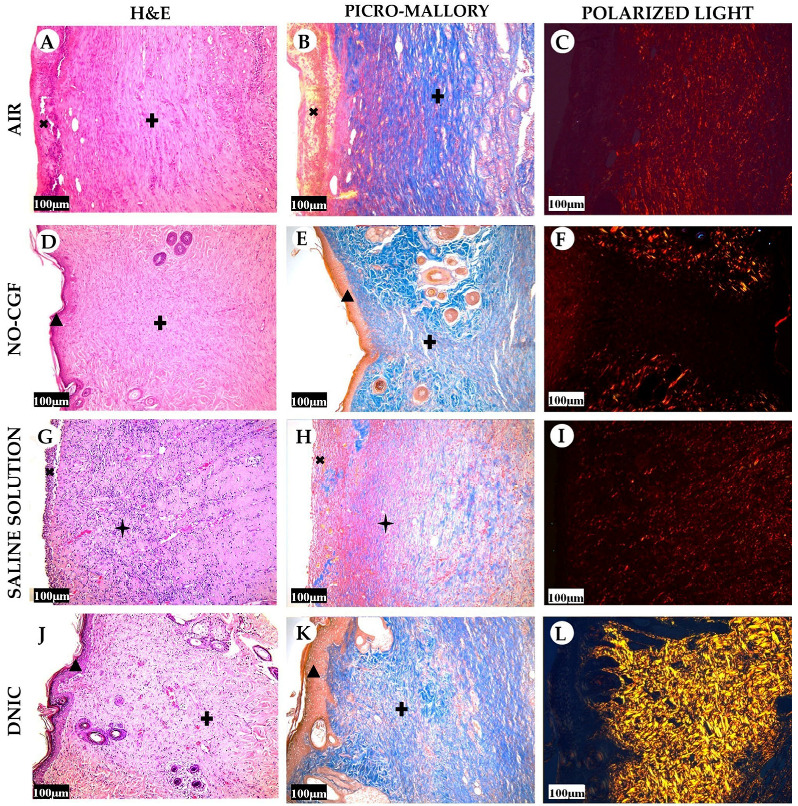
Histology of the wounds at post-operative day 21, hematoxylin and eosin staining (**A**,**D**,**G**,**J**), picro-Mallory staining (**B**,**E**,**H**,**K**) staining and polarized light microscopy (**C**,**F**,**I**,**L**); scale bar—100 μm. 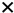
—fibrin clot, 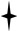
—granulation tissue, 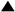
—epithelium, 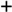
—fibrotic tissue.

**Figure A7:**
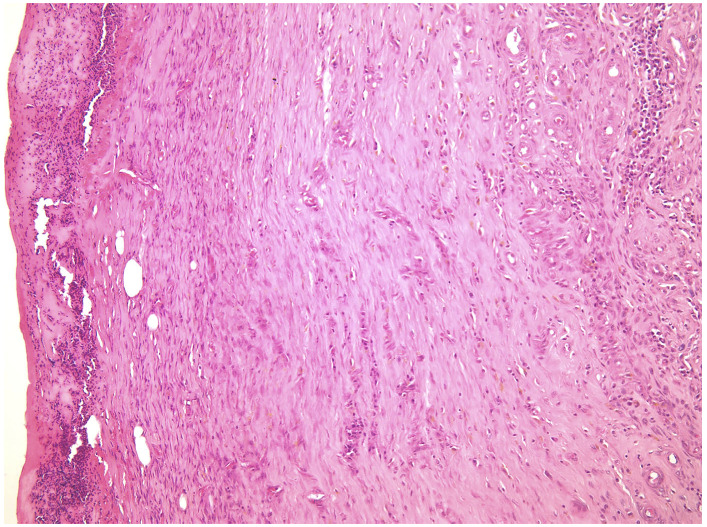
Histology of the wound treated with air at post-operative day 21, hematoxylin and eosin staining, magnification—10×.

On Day 21, in control group 1 (air), the epithelium covered only the wound’s edges. The wound’s bottom tissues were characterized by high cellularity (inflammatory infiltration, α-SMA and vimentin-positive cells) and collagen fiber content, clearly visible in picro-Mallory staining (Figure 13C,D, Figure 14C,D, Figure 15A,B and Figure A7). They appeared bright and red–yellow under polarized light (Figure 15C).

**Figure A8:**
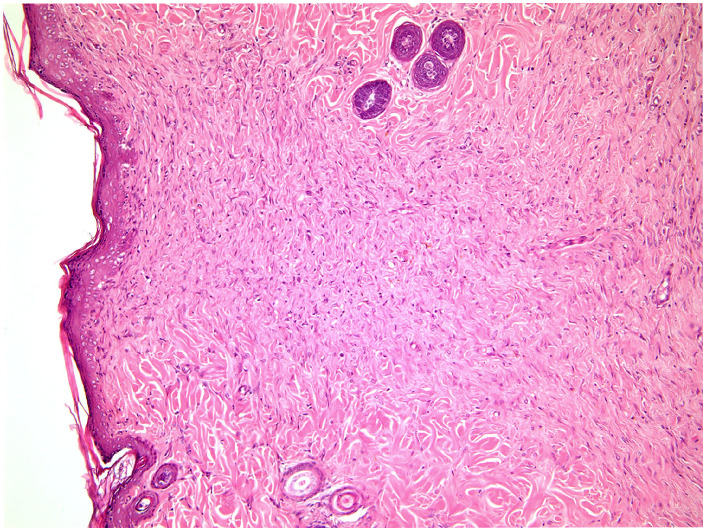
Histology of the wound treated with NO-CGF at post-operative day 21, hematoxylin and eosin staining, magnification—10×.

In the NO-CGF group, five out of six experimental animals had completely epithelized wound bottoms. The wound centers looked like mature granulation tissue with singular capillaries, vimentin-positive fibroblasts and thick collagen fibers with weak anisotropy (Figure 14G,H, Figure 15D–F and Figure A8). The area of α-SMA-positive cells was 19% smaller than in the air control group (*p* < 0.05) (Figure 9 and Figure 13G,H).

No wounds that were treated with saline healed completely. Immune cell infiltration and numerous vimentin-positive fibroblasts were observed in the majority of rats (Figure 14K,L and Figure 15G). The α-SMA expression index was 31% higher than in the DNIC group (*p* < 0.05) (Figure 9 and Figure 13K,L). Picro-Mallory staining revealed few collagen fibers, which had a weak birefringence in red color when studied by polarized light microscopy (Figure 15H,I). The other animals had less pronounced inflammatory findings and more mature granulation tissue with aligned collagen fibers.

In the DNIC group, the wound bottoms were epithelized. The collagen fibers and skin appendages regenerated, and the number of vimentin-positive cells decreased relative to Day 14 (Figure 14O,P and Figure 15J). The area of α-SMA-positive cells was 29.5% smaller compared to the air control group (*p* < 0.05) (Figure 9 and Figure 13O,P). Picro-Mallory staining revealed thick bundles of intertwined fibers (Figure 15K). Polarized light microscopy revealed strongly birefringent red collagen in the wound’s periphery dermis (Figure 15L).

The dynamic histological study revealed the maximum intensity of inflammation in the saline control group. NO-CGF application decreased inflammation and facilitated regeneration (fibroblast proliferation, angiogenesis, granulation tissue volume and maturity) during the wound healing period starting at the seventh day after surgery. The DNIC spray had little effect on inflammation but effectively increased the granulation tissue maturation at day 4 after surgery.

### 2.3. Transmission Electron Microscopy

**Figure 16:**
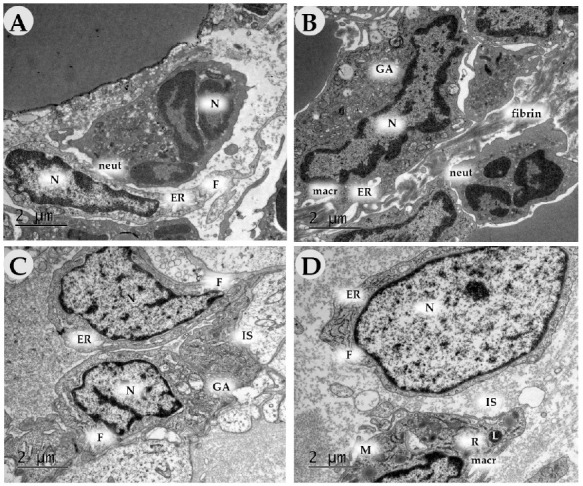
Transmission electron microscopy of the wound bottom of animals in the control (air (**A**), saline (**B**)) and experimental groups (NO-CGF (**C**), DNIC (**D**)) on day 3. Magnification ×15,000. neut—neutrophil, F—fibroblast, macr—macrophage, N—nucleus, M—mitochondria, GA—Golgi apparatus, L—lysosome, R—ribosome, ER—endoplasmic reticulum, IS—intermediate substance.

On Day 3 in the control groups, the inflammatory infiltrates mainly contained neutrophils with segmented nuclei, a dense cytoplasm and phagosomes (Figure 16A,B). In addition, we determined many macrophages with scalloped nuclei, numerous dense cytoplasmic granules (lysosomes) and long outgrowths of the plasma membrane (Figure 16B). Collagen fibers (apparently pre-existing operations) represented the extracellular matrix. Some of them did not have a specific cross-striation, which indicated their destruction. Young oval-shaped fibroblasts with a large nucleus with weakly condensed chromatin, Golgi apparatus, a poorly developed endoplasmic reticulum (ER) and a few free ribosomes predominated in the experimental groups (Figure 16A). In the experimental groups, macrophages predominated in the inflammatory infiltrate. The number of young and maturating (with developed granular ER) fibroblasts significantly increased (Figure 16C,D). Intermediate substances (glycosaminoglycans) represented the extracellular matrix; collagen production was absent.

**Figure 17:**
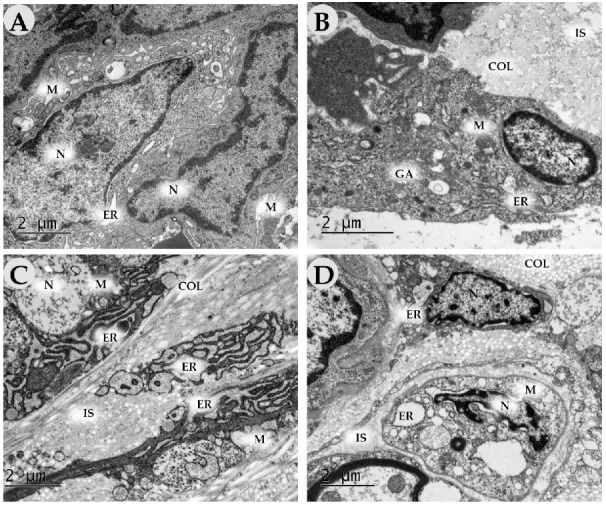
Transmission electron microscopy of the wound bottom of animals in the control (air (**A**), saline (**B**)) and experimental groups (NO-CGF (**C**), DNIC (**D**)) on day 7. Magnification ×15,000. N—nucleus, M—mitochondria, GA—Golgi apparatus, ER—endoplasmic reticulum, IS—intermediate substance, COL—collagen.

On Day 7, the number of fibroblasts increased in all groups. In the control groups, they had a less developed granular ER (low synthetic activity) and Golgi apparatus than in the experimental ones (Figure 17A–D). The extracellular matrix was comprised predominantly of fibrin and proteoglycan fibers, whereas, in the experimental groups, it contained collagen fiber bundles. In experimental group 2 (DNIC), we revealed numerous newly formed capillaries.

**Figure 18:**
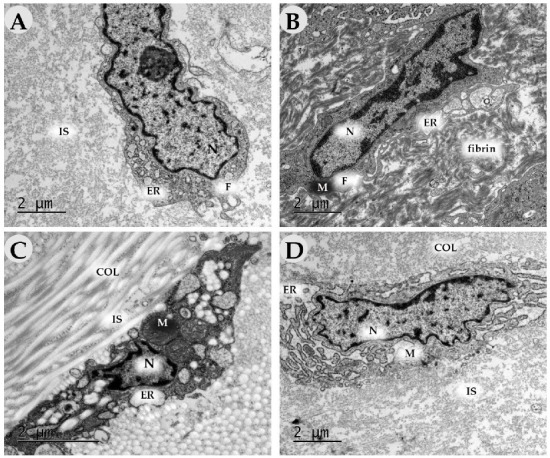
Transmission electron microscopy of the wound bottom of animals in the control (air (**A**), saline (**B**)) and experimental groups (NO-CGF (**C**), DNIC (**D**)) on day 14. Magnification ×15,000. F—fibroblast, N—nucleus, M—mitochondria, ER—endoplasmic reticulum, IS—intermediate substance, COL—collagen.

By Day 14, in the control groups (air, saline), there were fewer fibroblasts than in the experimental ones. The cells had a light cytoplasm, few organelles and a poorly developed endoplasmic reticulum, which suggests their immaturity. The extracellular matrix consisted of singular collagen fibers among intermediate substances (Figure 18A,B). In experimental groups (NO-CGF, DNIC), fibroblasts were orientated along dense bundles of collagen fibers (Figure 18C). The cells had large mitochondria. Dilated cisterns of granular ER were surrounded by ribosomes and polysomes, which indicates their high synthetic activity (Figure 18C,D).

The current histological study proved that NO-therapy effectively stimulates regeneration by activating fibroblast and myofibroblast proliferation, the growth and maturation of granulation tissue acceleration and NF-κß pathway modulation in the early stages of wound healing. However, NO-CGF had more prolonged effects on wound contraction and epithelialization and the decrease in inflammation than the DNIC spray.

## 3. Discussion

The Nobel Prize in Medicine and Physiology for NO molecule discovery (Furchgott R.F., Ignarro L.J., Murad F.) in 1998 initiated the bulk of studies investigating the properties and biological effects of NO and its compounds. Around the world, many researchers began to develop NO donors and methods for NO delivery [11,14,15,18,20].

The NO-therapy’s popularity is associated with the versatility of NO regulatory functions in normal conditions and pathology. This unique molecule penetrates through skin, cornea, mucous membranes and wound surfaces [3,6]. In the early stages of wound healing, NO reacts with superoxide and forms peroxynitrite. Its degradation products decrease the wound’s pH and enhance inflammation and bactericidal effects [4]. These effects are associated with multiple molecular pathways activations, in particular, NF-κβ. Nitric oxide and reactive nitrogen species can modify NF-κß activity in different ways [24]. In an inactive form, NF-κβ is located in the cytoplasm. When introduced to peroxynitrite, ultraviolet radiation, active radicals and other factors, it activates and translocates into the cell nucleus, stimulating activation of physiological and pathological processes, including the synthesis of pro-inflammatory cytokines, cell proliferation, apoptosis and the S-phase of the cell cycle, which is important for wound healing [25,26]. The NO molecule inhibits NF-κB activation by neutralization of pro-oxidative molecules and decreases the peroxynitrite formation at the end of the inflammatory phase of wound healing [27]. We calculated the NF-κβ activity as the ratio of the nuclear expression index to the cytoplasmic index. Our study demonstrated the activation of this protein in the macrophages of wounds treated with NO-therapy on the first week of wound healing. In these groups, the intensity of inflammation was significantly lower than in the control. This can be explained by NF-κβ stimulation of leukocyte phagocytic function [28]. The activation of this protein in wound fibroblasts can promote their proliferation as an NF-κß signaling pathway [26].

NF-κß dimer activation directly induces iNOS formation, a pro-inflammatory enzyme that is involved in the synthesis of endogenous NO in the wound in response to injury [29]. iNOS inhibition significantly reduces collagen synthesis, thus, NO-therapy can compensate for this effect [28]. As the concentration of NO increases, the modulation of NF-κß stops, the production of pro-inflammatory cytokines decreases and the antioxidant system is activated [26]. However, we did not observe the strong correlation between NF--κß and iNOS levels. This can be related to other signal molecules contributing to this enzyme expression, for instance, signal transducers and activators of transcription 3 (STAT3) and activator protein-1 (AP-1) [30,31].

The products of NO oxidation include an excess of nitrite anions, activated NO metabolism and synthesis of its endogenous donor, DNIC, in low pH conditions [2,32]. We identified significant differences in macrophage iNOS expression between groups in which wounds were treated with DNIC and saline on Day 7 after surgery. This observation did not correlate with the intensity of inflammation in these groups. Previously, it was considered that iNOS was one of the main enzymes involved in active radical formation and negatively affected cell survival. Later studies revealed that, besides irreversible damage to cell organelles, proteins and DNA, its low or moderate concentrations activate intracellular signaling pathways, stimulating the production of factors important for cell growth and proliferation [3]. An increase in iNOS expression in the macrophages impacts the inflammatory reaction through suppression of bacterial contamination and elimination of necrotic tissue at the early stages of wound healing [26]. An increase in the iNOS expression index in fibroblasts by Day 7 is important for the stimulation of collagen production in the healing wound.

In our study, we observed a significant intensification of fibroblast proliferation and vascularization in the groups with NO-therapy. According to the literature data, NO stimulates angiogenesis through cGMP-dependent cascade activation and regulates the fibroblast’s synthetic activity, even in low tissue concentrations [33,34]. Vimentin is one of the main fibroblast markers which is used to evaluate connective tissue growth. Persistence of vimentin-positive cells at the late period of wound healing is associated with scar formation [35]. Our results demonstrated that NO-therapy increased vimentin expression and fibroblast proliferation, accelerating the proliferation phase of wound healing during the first week after injury. On Day 4, most intensive morphological findings of regeneration were observed in the DNIC group. On Day 7 after injury, the maximum number of vimentin-positive cells was observed in wounds treated with NO-CGF.

These results indicate that both NO delivery approaches have strong pro-regenerative effects on fibroblasts. NO-therapy contributed to a decrease in the area with vimentin-positive cells, starting from the 14th day after surgery. By Day 21, the number of vimentin-positive cells in the experimental groups decreased faster than in the controls. It is possible that NO facilitates scar reduction, which is beneficial for the outcome of wound healing.

α-SMA is a marker of myofibroblasts, the cells which are important for wound contraction in the early stages of wound healing [36]. We detected a significant decrease in wound areas in the experimental groups on Day 7. In the later stages of wound healing, the number of myofibroblasts reduced because of apoptosis. It prevented excessive contraction of the wound edges and scarring [35,36]. NO-therapy-stimulated epithelialization of the wound defect and its healing without excessive contraction with a linear scar were achieved by Day 21.

**Figure 19:**
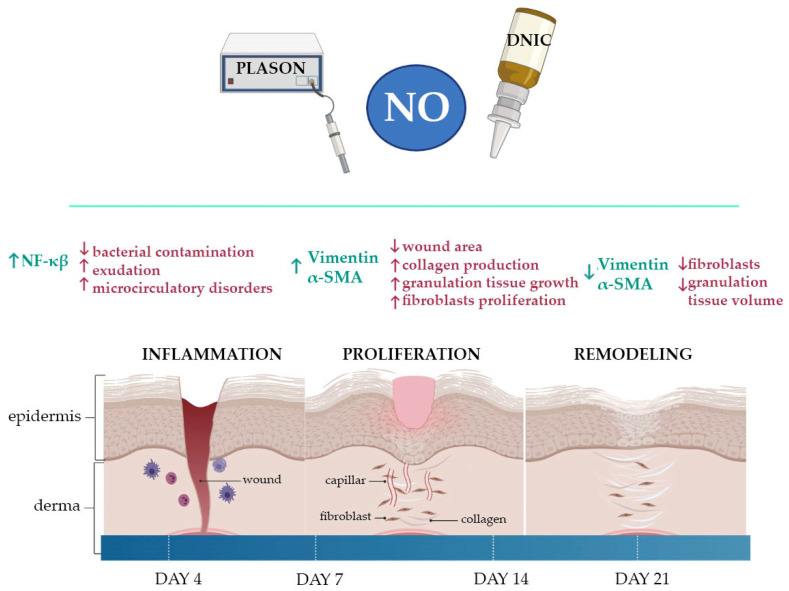
Nitric oxide’s effects in wound healing (created using Biorender.com).

In the present study, we proved that DNIC spray and NO-CGF application accelerated the reduction in wound areas in the early stages of wound healing (7 days) by stimulating myofibroblasts’ differentiation and the prevention of scar tissue remodeling at a later stage (21 days) because of a decrease in the number and activity of these cells (Figure 19).

A complex comparative study of skin wound healing effects of optimal doses of DNIC spray and NO-CGF did not reveal a significant difference in their impact on the molecular mechanisms of regeneration, judging by the expressions of NF-kB, iNOS, α-SMA and vimentin in wound tissues. We proved that the application of DNIC spray and NO-CGF at their optimal doses (0.02 and 1 mmol, respectively) accelerated the reduction in wound areas at the early stages of wound healing (7 days) by stimulating myofibroblast transdifferentiation and prevented scar tissue formation at a later stage (21 days) by decreasing the number and activity of these cells. This result allows us to conclude, definitively, that DNIC spray is more dosage effective as a pro-healing agent than NO-CGF considering differences in their molar doses.

The question then arises: what is the reason for this higher wound healing activity of DNIC spray? In other words, why does the incorporation of NO molecules into DNIC followed by their release from these complexes significantly improve their healing effect on wounds compared to NO molecules existing initially in the gas phase?

On the one hand, this composition protects molecular NO from various negative factors (for example, from the harmful effect of superoxide anions), and, on the other hand, does not impede its release from B-DNIC-GSH, which is necessary for the wound healing action of NO. The results of previous studies of B-DNIC-GSH and similar complexes with other thiol-containing ligands support this statement. First, the duration of the existence of DNIC with thiol-containing ligands in a solution, varying from several hours to several days depending on the nature of these ligands, is an indicator that the incorporation of NO into these complexes transforms it from a volatile gaseous agent into non-volatile water-soluble complexes [12]. As a result, when NO is introduced into the wound as part of DNIC, it escapes only after the disintegration of these complexes. When NO is delivered to the wound in gaseous form, a significant part of this agent can quickly evaporate from it without having a wound healing effect. Secondly, the experiments with protein-bound DNICs showed that the reaction constant of the free radical reaction between NO molecular ligands in these complexes and superoxide anions is three orders of magnitude lower than the similar reaction constant between these anions and NO in the gaseous phase [37]. If, however, we take into account that, upon entering animal tissues, low-molecular-weight M- and B-DNICs are almost completely (as a result of the transfer of iron-dinitrosyl fragments from these complexes to the thiol groups of proteins) converted into protein-bound DNICs, then the phenomenon of NO protection from the destructive effect of superoxide anions on it becomes crucial [12]. Thirdly, free NO molecules can bind to the heme groups of various proteins, concentrate in the lipid compartments of cells and tissues and, finally, be oxidized by oxygen and other oxidizing agents. Such behavior of NO molecules should obviously be largely prevented by the incorporation of NO into such very stable complexes as DNICs associated with thiol groups of proteins—protein-bound DNICs.

The subsequent transfer of NO from the complexes to the target of its biological action—the heme-containing protein guanylate cyclase (a key regulator of wound healing)—is carried out by low-molecular-weight DNICs that intercept the iron–dinitrosyl group from the protein-bound DNICs with subsequent transfer of NO to the heme group of guanylate cyclase. It is clear that this transfer is determined, primarily, by the high affinity of this group for NO compared to the iron atom in DNIC and, secondly, by the selective binding of DNIC to the guanylate cyclase apo protein.

## 4. Materials and Methods

### 4.1. NO-Containing Gas Flow

We used the Plason device serial number 450, manufactured by Center BMSTU, LLC (Moscow, Russia). It was equipped with a standard manipulator with an outlet channel diameter of 1.4 mm. The distance to the wound surface was 120 mm. The NO-containing gas flow had the following axial parameters: temperature—50 °C, velocity—5 m/s, NO concentration—500 ppm, NO_2_ concentration—30 ppm, NO consumption—1.7 mg/s and mass flow rate of NO_2_—0.15 mg/s. The average NO consumption density during the 120 s long wound treatments was ~0.25 mg/(s∙cm^2^); the mass dose of NO supplied to the wound was 90 mg [8,20].

### 4.2. DNIC Containing Spray

DNIC with glutathione was obtained according to the procedure described by Borodulin and Vanin [38]. Solutions with a concentration of 50 mg/L were prepared by dissolving lyophilized DNIC-glutathione powder in sterile PBS at 25 °C. The spraying dosage of DNIC was 16.6 mg of the active substance per 1 cm^2^. Then, they were aliquoted into 4 plastic spray bottles of 50 mL (BX202005C, Ningbo Beixuan International Trading Co., Ltd., Ningbo, China) and frozen at −70 °C until application. The sprayability, stability of the spray and dosage were tested as described in our previous report [19].

### 4.3. Animal Studies

The experiment on 96 Wistar rats (males, 180–220 g) was approved by the Local Ethical Committee of Sechenov University (Protocol 15-19/25 November 2019). The animals were kept under the standard vivarium conditions of one animal per cage and were provided with complex granulated laboratory chow and constant access to water.

Full-thickness skin wounds were modeled in 96 Wistar rats. For anesthesia, a 25% urethane solution (Sigma, St. Louis, MI, USA) intraperitoneal injection was used at the dose of 80 mg of active ingredient per 100 g of animal weight. In the interscapular space, a circle with a diameter of 8–10 mm was excised to the fascia propria. We used Teflon rings with an inner diameter of 195 mm for the first 4 days after surgery. They prevented early wound contraction, which is usually more rapid than epithelialization in rats [39]. Moreover, the Teflon rings proved to be useful because of the elasticity of rat skin and its adhesion to the propria fascia. Furthermore, this ring was useful for treatment dosage control. It was covered with a perforated plastic wrap to prevent drying, external contamination and early wound contraction. All the wounds had a standard area of 3 cm^2^.

**Figure 20:**
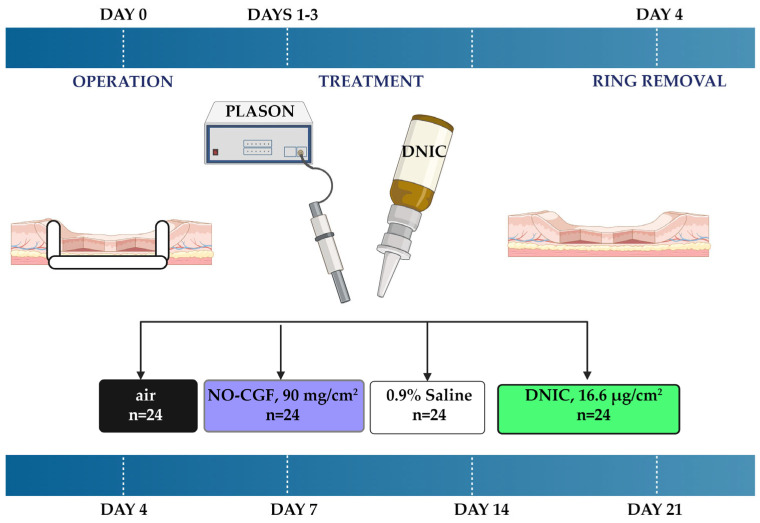
Experimental design (created using Biorender.com).

On postoperative days 1, 2 and 3, the animals were injected with ZOLETIL 100 (Virbac France, Carros, France) at a dose of 300 µg of the active ingredient per 100 g of the animal’s body weight for sedation. Then, the protective film was removed from the ring, and the wounds were assessed for signs of inflammation: edema, hyperemia, exudation and infiltration of the surrounding tissues. After that, the wounds were treated with atmospheric air (*n* = 24, air control group), NO-CGF (*n* = 24, NO-CGF group), sterile 0.9% Saline Solution (*n* = 24, saline control group) or DNIC spray (*n* = 24, DNIC group) (Figure 20). Six animals from every study group were sacrificed on days 4, 7, 14 and 21 after the operation, and the wound tissues were excised for histological analysis. Small pieces of central parts of the samples were excised for transmission electron microscopy on days 4, 7 and 14.

### 4.4. Wound Area Measurement

At 7, 14 and 21 days of the experiment, the contours of the wounds were outlined with a permanent marker on polyethylene film. Then, the figures were visualized and measured in micrometers using the Leica Application Suite, version 4.9.0 (Wetzlar, Germany). The values were recalculated in cm^2^ using a ruler scale bar.

### 4.5. Histological Study

The animals were sacrificed on days 4, 7, 14 and 21 after the operation, and the wound tissues were excised for histological analysis. Then, 4 μm-thick sections of the formalin-fixed, paraffin-embedded tissue samples were stained with hematoxylin and eosin (H & E) (BioVitrum, St. Petersburg, Russia), picrosirius red (BioVitrum, St. Petersburg, Russia), toluidine blue (BioVitrum, St. Petersburg, Russia) and by picro-Mallory (BioVitrum, St. Petersburg, Russia). A LEICA DM4000 B LED microscope equipped with a LEICA DFC7000 T digital camera running under the LAS V4.8 software (Leica Microsystems, Wetzlar, Germany) was used for the examination of the samples. Sections stained with picrosirius red were examined by polarized light microscopy. The panels were composed of microphotographs of central parts of the wounds for standardized assessment of wound healing.

### 4.6. Morphometrical Analysis

To evaluate morphological findings of inflammation (exudation, contamination, immune cell infiltration, microcirculatory disorders) and regeneration (angiogenesis, fibroblast proliferation, volume and maturity of granulation tissue), we used a 0 to 4 score system (0—absence of the findings, 4—maximum intensity of the findings).

The granulation tissue layer thickness was measured perpendicular to the epithelial layer in each sample at 5 random sites located at least 400 μm apart from each other. The measurements were made using the Leica Application Suite, version 4.9.0 (Leica Microsystems, Wetzlar, Germany).

### 4.7. Immunohistochemical Study

Sections, 4 μm thick, of the formalin-fixed, paraffin-embedded tissue samples were deparaffinized, underwent heat-induced epitope retrieval (5% Trilogy 20× solution (Cell Marque, Rocklin, CA, USA), 30 min in 80 °C water bath), incubation in 3% H_2_O_2_ for 15 min and block with Background Block (Cell Marque, Rocklin, CA, USA). Vimentin (ab92547, Abcam, Cambridge, UK, diluted 1:400) was chosen as a marker of fibroblasts, and α-SMA (ab5694, Abcam, Cambridge, UK, diluted 1:400) was chosen as a marker of myofibroblasts. We also studied the expressions of the NF-kß (ab16502, Abcam, Cambridge, UK, diluted 1:200) and iNOS (PA1-036, Invitrogen, Waltham, MA, USA, diluted 1:200), the enzyme involved in the synthesis of NO in the wound. After overnight incubation, HRP-conjugated secondary goat antibodies (G-21040, Invitrogen, Waltham, MA, USA, diluted 1:1000) and diaminobenzidine (DAB, 34002, Thermo Fisher Scientific, Waltham, MA, USA) were applied. Slides were counterstained with hematoxylin (Biovitrum, St. Petersburg, Russia).

For evaluation of IHC markers’ expression in the wounds, five different fields of view of each section were photographed at ×400 magnification and examined by two blinded pathologists using the formula: Expression Index (E) = (Positive Cells/Total Cells) × Label Intensity. The Label Intensity was determined by a pattern of IHC reaction on the microphotograph, where “0”—no reaction, “1”—weakly positive staining of singular cells, “2”—positive staining of less than 50% of cells and “3”—positive staining of more than 50% of cells. We evaluated the expression of iNOS and NF-kß separately in fibroblasts (spindle shaped cells with centrally placed oval nucleus) and macrophages (characterized by round shape and large elongated nucleus). Since the role of NF-kß depends on the subcellular localization, the activity of this marker (A) was calculated using the formula: A = E1/E2, where E1 is the expression index in the nucleus and E2 is the expression index in the cytoplasm.

### 4.8. Transmission Electron Microscopy

The samples were fixed in 4% glutaraldehyde (Pan Real, Spain) in 0.1 M phosphate buffer, pH 7.2–7.4 (Amresco, Boise, ID, USA; pH 7.2–7.4) at 4 °C for a day and postfixed in 1% OsO_4_ for 2 h. They were washed with a buffer solution, dehydrated in a graded series of ethanol-water washes and 2% uranyl acetate for a night and embedded in Epon (Epon 812, Fluka, Buchs, Germany). Cross-sections from central areas of the wounds were 1 μm in thickness. They were prepared on pyromitom for spatial orientation of the material and stained with 1% methylene blue (Biovitrum, St. Petersburg, Russia). Ultrathin cross-sections were poststained with 2% uranyl acetate (541-09-3, VWR International, Radnor, PA, USA) and alkaline lead citrate (Reynolds, 1963) and observed in a JEOL JEM-1011 transmission electron microscope (JEOL, Tokyo, Japan) at magnifications of 8000×, 15,000×, and 25,000×.

### 4.9. Statistical Analysis

Statistical analysis of the data was performed with the standard software package of GraphPad Prism, version 8.00 for Windows (GraphPad Software, Inc., San Diego, CA, USA). The distribution of the quantitative data was checked by Shapiro–Wilk’s normality test. The intergroup differences were analyzed using the two-way ANOVA followed by Tukey’s multiple comparison test. The differences in the histological scores were evaluated by a Kruskal–Wallis test followed by Dunn’s multiple comparison test. The statistical analysis results were presented as interleaved bar graphs of the mean values (for quantitative data) and standard deviations of the mean (SD) or median values (for collagen maturity, inflammation and regeneration indexes) and 95% confidence interval; *p*-values ≤ of 0.05 were considered statistically significant.

## 5. Conclusions

Our research demonstrated that both methods of NO delivery accelerated wound healing by having an impact on the same mechanisms of regeneration. NO-therapy reduced inflammation and promoted fibroblast proliferation, growth of granulation tissue and wound epithelization. DNIC spray application was particularly effective in stimulating the early wound healing process during the first 4 days after trauma. Further intensity of wound healing differed slightly. It should be taken into account that the content of the effective dose of NO in NO-CGF is 25 times higher than in DNIC.

## Data Availability

The relevant data generated and (or) analyzed in the current study are available from the corresponding author upon reasonable request.
